# Evaluation of Admission Chest X-Ray Findings in Patients With Respiratory Infection During the COVID-19 Pandemic

**DOI:** 10.7759/cureus.18114

**Published:** 2021-09-20

**Authors:** Dimitrios Velissaris, Maria Lagadinou, Themistoklis Paraskevas, Eleousa Oikonomou, Vasileios Karamouzos, Fotios Sampsonas, Markos Marangos, Menelaos Karanikolas

**Affiliations:** 1 Department of Internal Medicine, University Hospital of Patras, Patras, GRC; 2 Intensive Care Unit, University Hospital of Patras, Patras, GRC; 3 Department of Respiratory Medicine, University Hospital of Patras, Patras, GRC; 4 Department of Anaesthesiology, Washington University School of Medicine, Saint Louis, USA

**Keywords:** thoracic radiography, chest x-ray (cx-ray), covid-19, pneumonia, emergency medicine

## Abstract

Aims: To evaluate the prevalence of X-ray findings in hospitalized patients requiring hospitalization with suspected Coronavirus disease 2019 (COVID-19) infection and potential differences in the laboratory values and clinical outcomes related to the presence of abnormal chest X-ray (CXR) findings.

Methods:* *A total of 117 patients suspected of COVID-19 pneumonia and hospitalized with symptoms of lower respiratory tract disease were included in this study. Patients were divided into subgroups according to COVID-19 diagnosis and statistical comparisons were made according to CXR findings.

Results:* *In our cohort, CXR abnormalities were more common in patients with confirmed COVID-19 diagnosis and were associated with increased mortality. Patients with abnormal chest X-rays had a significantly lower PaO_2_/FiO_2_ ratio both in the COVID-19 and non-COVID-19 groups.

Conclusion: CXR is a routine examination in all patients with symptoms of lower respiratory tract disease and its findings relate to in-hospital mortality and PaO_2_/FiO_2_ ratio. Thus, it can be a significant measure of disease severity, especially in resource restrained settings and emergency situations such as the COVID-19 pandemic.

## Introduction

Coronavirus disease 2019 (COVID-19) has been declared a pandemic by the World Health Organization (WHO) since March 2020. After the partial retreat of the first wave, a second wave with an increased number of infected people occurred worldwide since October 2020. The respiratory system is the most frequently affected by severe acute respiratory syndrome Coronavirus 2 (SARS-CoV-2) and patients present with a wide spectrum of symptoms, from mild illness to more severe disease such as acute respiratory distress syndrome (ARDS) requiring intensive care unit (ICU) admission [1,2]. Reported mortality rates differ significantly among studies, ranging from 3% to 15%, probably due to different sample sizes, origin, diagnostic criteria, applied tests among investigations, and therapeutic approaches. In the age of COVID-19, suspected cases are often hospitalized in special wards and respiratory support must begin before the results of COVID-19 reverse transcription-polymerase chain reaction (RT-PCR) are available.

Chest X-ray (CXR) is a routine examination in such patients, but its clinical value is often underestimated. In this setting, CXR might play a prognostic role, while its diagnostic value seems uncertain due to the low specificity of its findings in the differentiation between the different infectious agents.

The aim of this study was to evaluate the prevalence of X-ray abnormalities in hospitalized patients with suspected COVID-19 and potential differences in the laboratory values and clinical outcomes related to the presence of abnormal CXR findings.

## Materials and methods

Patients

This prospective observational study enrolled patients with signs and symptoms of lung lower respiratory tract disease hospitalized in the Department of Internal Medicine of a tertiary academic medical center in western Greece. Patients under 18 years old and patients who refused consent were excluded from this study. The patients were treated in a modified Ward with precautions due to the possible transmission of SARS-CoV-2 and were divided into two groups according to the polymerase chain reaction (PCR) results for SARS-CoV-2 infection. The study period was from January 1, 2021 until January 31, 2021, during the period of the second wave of the current COVID-19 pandemic in Greece. The two groups consisted of 30 COVID-19 patients and 87 non-COVID-19 patients. Clinical examination and laboratory screening with inflammatory markers (white cell blood count (WBC), total number of lymphocytes, C-reactive protein (CRP), lactate dehydrogenase (LDH), d-dimers, ferritin, fibrinogen) were undertaken in all patients immediately upon admission. Radiographic findings via CXR evaluation on admission were also assessed. All patients gave consent for participating in the study and the Ethics Committee of the General University Hospital of Patras approved the protocol (Protocol Number: 102/09-03-2021).

Statistical analysis

Patient data were analyzed by using Chi-square, Fisher’s Exact, and Mann Whitney U tests, as appropriate. Results are presented in tables as median (25th, 75th percentile). P-values less than 0.05 indicate statistical significance. All data analysis was conducted using the SPSS statistical software package (IBM Corp. Released 2019. IBM SPSS Statistics for Windows, Version 26.0. Armonk, NY: IBM Corp).

## Results

Demographic and laboratory values in patients with COVID-19 versus patients without COVID-19 are shown in Table 1. COVID-19 positive patients had a significantly lower number of total WBC, lower absolute number of lymphocytes, lower levels of d-dimers, and increased plasma levels of ferritin, LDH, and creatine phosphokinase (CPK). In addition, these patients also had a significantly lower PO_2_/FiO_2 _ratio (p=0.004) and longer length of hospital stay (p=0.02) when compared to non-COVID-19 patients, but there was no significant difference with regards to mortality.

**Table 1:** Demographic and Laboratory data in COVID-19 positive versus COVID-19 negative patients. Demographic and laboratory values in both groups of patients (with COVID-19 vs. without COVID-19). Values are listed as median (25th, 75th percentile). WBC: white blood cells, CRP: C-reactive protein, LDH: lactate dehydrogenase, CPK: creatine phosphokinase.

	COVID-19 positive (n=30)	COVID-19 negative (n=87)	P-value
Age (years)	65.5 (55,74)	69 (55, 80)	0.473
Body mass index	27 (25, 31)	25 (23, 31)	0.127
Gender (M/F)	20/10	56/31	0.82
WBC (K/mL)	6105 (4310, 7710)	9060 (7485, 14,560)	<0.001
Total lymphocytes (K/mL)	840 (520, 1418)	1290 (750, 2135)	0.012
CRP (mg/dL)	6.18 (1.86, 10.18)	6.02 (2.10, 13.50)	0.528
Lactate (mmol/L)	1.15 (0.80, 1.40)	1.20 (0.80, 1.80)	0.625
Fibrinogen (mg/dL)	509 (404, 682)	569 (448, 661)	0.577
D-Dimers (ng/mL)	1057 (460, 1890)	1270 (690, 2725)	0.257
Ferritin (μg/L)	654.5 (336.5, 937)	149.5 (56, 384)	<0.001
LDH (U/L)	361 (238, 502)	241, (201.5, 309.5)	0.006
CPK (U/L)	102.5 (71, 149)	71 (47, 128)	0.032
PO_2_/FiO_2_	281.7 (174, 390)	379.8 (288.6, 441.2)	0.004
Length of hospital stay	6.5 (4, 13)	3 (2, 6)	0.002
Chest X-ray abnormalities (presence/absence)	25/5	37/50	<0.001
Outcome (dead/alive)	6/24	8/79	0.116

CXR findings

Twenty-five of 30 (83.33%) COVID-19 positive patients had abnormal findings in the CXR (bilateral lesions), compared to 37 of 87 (42.53%) in COVID-19 negative patients, and the difference was highly significant (p<0.001).

In our cohort, there was a significant increase in mortality (p=0.0014) and a decrease in the P/F ratio (p<0.01) in patients with CXR abnormalities.

We further analyzed the data, by dividing COVID-19 positive patients into two sub-groups based on the presence or absence of abnormal CVR findings. This analysis showed that mortality was 24% (6 of 25) in patients with an abnormal CXR vs. 0% mortality (0 of 5) in patients with normal CXR. Although mortality is higher in those with abnormal CXR, the difference did not reach significance (P=0.553), likely due to the small sample size. Length of hospital stay was not different among the two sub-groups. However, patients with COVID-19 who had abnormal CXRs had significantly higher CRP, lactate, and LDH levels and significantly lower PO_2_/FiO_2_ compared to COVID-19 positive patients without CXR abnormalities. These findings are presented in Table 2.

**Table 2:** Demographic and laboratory values in COVID-19 positive patients with versus without chest infiltrates on CXR COVID-19 patients based on the presence of chest infiltrations. Values are presented as median (25th, 75th percentile). CXR: chest X-ray, WBC: white blood cells, CRP: C-reactive protein, LDH: lactate dehydrogenase, CPK: creatine phosphokinase.

	COVID-19 patients with abnormal CXR (n=25)	COVID-19 patients with normal CXR (n=5)	P-value
Age (years)	66 (55.5, 79)	55 (37.5, 70.5)	0.251
BMI	28 (24, 32)	27 (26.25, 27.75)	0.818
Gender (M/F)	16/9	4/1	0.640
WBC (K/mL)	6110 (4585, 7665)	6100 (2790, 11,250)	1.000
Total lymphocytes (K/mL)	830 (510, 1404)	930 (540, 1835)	0.481
CRP (mg/dL)	8.78 (3.41, 12.63)	1.01 (0.38, 2.10)	0.003
Lactate (mmol/L)	1.2 (0.8, 1.9)	0.8 (0.45, 1.1)	0.042
Fibrinogen (mg/dL)	521.5 (405, 682)	474 (381.5, 616.5)	0.482
D-Dimers (ng/mL)	1130 (580, 1910)	380 (315, 2120)	0.136
Ferritin (μg/L)	677 (324, 978)	432 (229, 1337)	0.727
LDH (U/L)	388 (241, 569)	184 (159, 371.5)	0.042
CPK (U/L)	100 (70, 151.5)	118 (75, 174.5)	0.787
PO_2_/FiO_2_	260 (156, 342)	400 (397, 464)	<0.001
Length of hospital stay	7 (4, 14)	2 (2, 9)	0.136
Outcome (dead/alive)	6/19	0/5	0.553

In patients with abnormal CXRs who tested negative for SARS-CoV-2, mortality was 18.9% (7 of 37) in patients with abnormal CXR, compared to 2% (1 of 50) in patients without CXR infiltrations and the observed difference was highly significant (P=0.009). However, there was no significant difference in hospital length of stay. Demographic and laboratory results in the COVID-19 negative patients with versus without abnormal CXR findings are presented in Table 3. P/F ratio significantly differed between the two groups (p<0.01).

**Table 3:** Demographic and laboratory values in COVID-19 negative patients with and without chest infiltrates on CXR Non-COVID-19 patients based on the presence of chest infiltrations. Values are presented as median (25th, 75th percentile). CXR: chest X-ray, WBC: white blood cells, CRP: C-reactive protein, LDH: lactate dehydrogenase, CPK: creatine phosphokinase.

	Non-COVID-19 patients with abnormal CXR (n=37)	Non-COVID-19 patients with normal CXR (n=50)	P-value
Age (years)	71 (58, 80)	66.5 (53,80)	0.275
BMI	25.5 (23, 31)	25 (2331.5)	0.775
Gender (M/F)	27/10	29/21	0.149
WBC (K/mL)	10,640 (7490, 15,675)	8515 (7352, 13,647)	0.238
Total Lymphocytes (K/mL)	1430 (800, 2050)	1245 (685, 2207)	0.680
CRP (mg/dL)	7.06 (1.95, 13.5)	4.50 (2.1, 14.9)	0.990
Lactate (mmol/L)	1.10 (0.80, 1.70)	1.29 (0.80, 1.83)	0.419
Fibrinogen (mg/dL)	568.5 (372, 684)	569.5 (376, 648)	0.188
D-Dimers (ng/mL)	1400 (585, 2510)	1265 (747.5, 3205)	0.843
Ferritin (μg/L)	138 (50, 338.5)	186 (59, 482.5)	0.420
LDH (U/L)	251 (200.5, 306.5)	235.5 (200, 323)	0.734
CPK (U/L)	93 (51, 176)	66.5 (42, 125)	0.139
PO_2_/FiO_2_	309 (192.5, 392.4)	400 (376, 452)	<0.001
Length of Hospital Stay	4 (2,7)	3 (2, 5.5)	0.747
Outcome (dead/alive)	7/30	1/49	0.009

## Discussion

In this study, we report that in the setting of hospitalized patients with symptoms of lower respiratory tract infection suspected with COVID-19, patients with a diagnosis of COVID-19 have a significantly higher proportion of abnormal CXR, compared to those with a diagnosis of non-COVID-19 pneumonia. In addition, we found that irrespective of COVID-19 diagnosis, patients with an abnormal CXR have a lower P/F ratio, which corresponds to increased disease severity. Lastly, in our cohort, there was a significant difference in laboratory biomarkers (CRP, LDH, lactate) in patients with abnormal CXR compared to patients without chest infiltrates, only among the patients with COVID-19.

CXR is a fast and cheap diagnostic tool, which can be performed in the majority of patients even in the emergency department with the help of portable X-ray units. Modified radiological scores have been used to evaluate the prognosis of COVID-19 patients [3,4], but due to the lack of specific findings in COVID-19 patients, its use is limited [5]. The prognostic capabilities of CXR and its use as a triage tool in non-severe COVID-19 patients are highlighted in a study by Sempere-Gonzales [6]. Computed tomography is being used to identify abnormalities in patients with COVID-19, but it is not available in all clinical settings and is costly compared to CXR [7,8]. Nevertheless, computed tomography findings are related to clinical severity and prognosis, thus making it a useful supplemental tool for patients’ evaluation [9,10]

In COVID-19 patients, several changes in commonly used laboratory parameters are described. In the study by Broman, IL-6 and CRP were the strongest predictors of severity in hospitalized patients with COVID-19 [11], while in another study, it was shown that abnormal levels of neutrophil to lymphocyte ratio (NLR), LDH, d-dimers, CRP, fibrinogen, and ferritin can be identified early on admission COVID-19 and can predict the severity of the disease [12]. In our study, we identify the presence of combined abnormalities of common laboratory parameters and CXR infiltrations in COVID-19 patients. Predictive scores that integrate easily available radiological and laboratory markers could be of great significance in resource-constrained settings and emergency situations [13].

The main limitation of our study is the small number of patients enrolled and that it is a single-center study. Additionally, the exact pathogen was not identified in patients with non-COVID-19 pneumonia, but they were considered and treated as bacterial pneumonia. On the contrary, there were no signs of superimposed bacterial infection in any of the COVID-19 patients in our cohort.

## Conclusions

In our study, the presence of abnormalities in the CXR of COVID-19 and non-COVID-19 patients was associated with reduced P/F ratio and increased in-hospital mortality. As CXR is a routine examination in all patients with symptoms of lower respiratory tract infection, it can be a significant measure of disease severity, especially in resource restrained settings and emergency situations. Multi-center studies are needed to identify the prognostic value of the combined presence of abnormal laboratory parameters and CXR abnormalities in COVID-19.
